# Editorial: Coping With Pollution – the Effects of Environmental Contaminants on Plant Growth and Physiology

**DOI:** 10.3389/fpls.2021.740802

**Published:** 2021-08-23

**Authors:** Marcelo Pedrosa Gomes, Eduardo Gusmão Pereira, Bao-Sheng Qiu, Philippe Juneau

**Affiliations:** ^1^Laboratório de Fisiologia de Plantas sob Estresse, Departamento de Botânica, Setor de Ciências Biológicas, Universidade Federal do Paraná, Curitiba, Brazil; ^2^Laboratório de Fisiologia do Estresse Abiótico, Instituto de Ciências Biológicas, Universidade Federal de Viçosa, Florestal, Brazil; ^3^Hubei Key Laboratory of Genetic Regulation and Integrative Biology, School of Life Sciences, Central China Normal University, Wuhan, China; ^4^Ecotoxicology of Aquatic Microorganisms Laboratory, EcotoQ, GRIL, TOXEN, Department of Biological Sciences, Université du Québec à Montréal, Montréal, QC, Canada

**Keywords:** emerging contaminants, phytoremediation, climate changes, trace element, xenobiotic

Environmental contamination as a consequence of anthropogenic activities has become a global concern. Legislative changes and polices on a global scale surrounding soil, water, and atmospheric contamination require continued attention and development of new approaches to mitigate damage. Urbanization, mining, industry, and certain agricultural practices, as well as improper waste disposal, are largely responsible for undesirable release of hazardous compounds to the environment. These pollutants, such as trace-elements, can negatively affect plant growth and physiology, including those of agricultural interest, causing adverse ecological and economic outcomes. Moreover, the response of plants to pollutants and contaminants requires more investigation. It is important to better elucidate the uptake, accumulation in the food chain, and the plant's capability to resist and “reclaim” pollutants from contaminated environments. In this context, the identification of tolerance and sequestration mechanisms in plants to hazardous compounds can help the development of environmentally friendly technologies, i.e., phytoremediation. Moreover, such information may support molecular approaches to increase plant tolerance to contaminants, such as plant transformation and genetic modifications. This Research Topic aimed to gather knowledge on advanced plant responses to environmental contamination.

Metal contamination is of particular interest for studies due to their continuous insertion and toxicity to environment. Using the green eukaryotic model microalga *Chlamydomonas reinhardtii* Dangeard, Du et al. revealed responses to lead (Pb), using a comprehensive approach, including physiological, genomic, and transcriptomic analyses. It was advanced that changes in protein folding, and endoplasmic reticulum-mediated protein quality control mechanism are influenced by Pb.

The effects of atmospheric contamination by metals such as mercury (Hg) were also studied by Sun et al. using the Hg-bioindication epiphytic species *Tillandsia usneoides* L. The species showed strong tolerance to the metal and, in addition, the activity of superoxide enzymes as well as the concentrations of glutathione (GSH) and metallothionein were stated as biomarkers and indicators of hormesis in response to Hg stress in *T. usneoides*.

The regulation of miRNA-mediated response to chromium (Cr) has been little studied so far. In a study based on high-throughput microRNA sequencing, Nie et al. documented a total of 104 conserve miRNAs and 158 non-conserved miRNA in *Miscanthus sinensis* Andersson, exposed to the metal. The authors identified that three miRNA (miR167a, novel_miR15, and novel_miR22) and their targets being involved in Cr transportation and chelation in plants and demonstrated the involvement of four miRNA (miR156a, miR164, miR396d and novel_miR155) in biochemical metabolism and detoxification of Cr, establishing the critical role of miRNAs in plant responses to metals.

In *Brassica napus* L., Jung et al. evidenced the link between ascorbate (AsA) redox status and GSH and/or NADPH redox ratios through the AsA-GSH-NADPH cycle and suggested the exogenous application of AsA as a possible alternative to alleviate Cd toxicity in oilseed rape seedlings grown in soils contaminated by this metal.

Soil salinization is one of the most limiting aspects for plant growth. After being observed the expression of tyrosine decarboxylase gene *MdTyDc* in plants of *Malus domestica* Borkh. under alkaline Liu et al. demonstrated that this gene plays an important role in apple plants' resistance to salinity. The overexpression of *MdTyDc* enhanced photosynthesis by improving root activity, the activity of enzymes related to nitrogen metabolism and promotion of N absorption. This gene was noted to enhance alkaline tolerance and performance of apple plants under salinity conditions.

Kintlová et al. performed a genome-wide transcriptome analysis on barley plants (*Hordeum vulgare* L.) treated with increased concentrations of cadmium (Cd). Authors identified the deregulation of genes related to reactive oxygen species metabolism, cell wall formation and maintenance, ion membrane transport and stress responses. For the very first time, it was identified four different copies of the PLANT CADMIUM RESISTANCE 2 (HvPCR2) genes, responsible for metal detoxification in plants, indicating multiplication of the PCR2 gene in plants. In turn, in their contribution Zhu et al. observed the positive effects of the overexpression of the phytochelatins (PC) gene *BnPCS1* in ramie [*Boehmeria nivea* (L.) Gaud.] submitted to Cd stress. Overexpression lines of *BnPCS1* showed great Cd-tolerance, as evaluated by plant growth, H_2_O_2_ concentrations and lipid peroxidation, and higher accumulation and translocation of the metal from the root to shoots, indicating the *BnPCS1* as a new gene resource for phytoremediation of Cd-contaminated soils.

We are grateful to all contributors for presenting such a variety of aspects of different complexity levels from molecular to physiological responses of plants to environmental contaminants. It also became clear the emerging role of molecular biology to be applied both for understanding plant responses but also to enhance plant performance on environmental remediation programs. The contributions from this Research Topic also highlighted the remaining knowledge gaps, among them are (not exclusive):

- The interactive effects of different contaminants on plants
- The interactive effects of contaminants and changes in the environment arising from climate change (such as increasing temperatures, irregular rain patterns, and adverse light conditions)
- The physiological markers for environmental monitoring and attest the capability of the plants to resist and “reclaim” pollutants from contaminated environments;
- Field studies regarding the use of plants to reclaim contaminants from contaminated environments;
- The effects of contaminants on crop production and food safety.

In conclusion, in a scenario of climate changes, priorities for future work on environmental contaminants and their effects should focus on investigating their isolated and combined effects with other chemicals and environmental abiotic and biotic conditions.

## Author Contributions

All authors listed have made a substantial, direct and intellectual contribution to the work, and approved it for publication.

## Conflict of Interest

The authors declare that the research was conducted in the absence of any commercial or financial relationships that could be construed as a potential conflict of interest.

## Publisher's Note

All claims expressed in this article are solely those of the authors and do not necessarily represent those of their affiliated organizations, or those of the publisher, the editors and the reviewers. Any product that may be evaluated in this article, or claim that may be made by its manufacturer, is not guaranteed or endorsed by the publisher.

